# Language-Concordant Care: a Qualitative Study Examining Implementation of Physician Non-English Language Proficiency Assessment

**DOI:** 10.1007/s11606-023-08354-6

**Published:** 2023-08-24

**Authors:** Maria Esteli Garcia, Mia Williams, Sunita Mutha, Lisa C. Diamond, Jane Jih, Margaret A. Handley, Sarita Pathak, Leah S. Karliner

**Affiliations:** 1 Division of General Internal Medicine, Department of Medicine, Multiethnic Health Equity Research Center, University of California, 1701 Divisadero St. Room 536, San Francisco, CA 94143-1731 USA; 2 Department of Epidemiology and Biostatistics, Implementation Science Training Program, UCSF, San Francisco, CA USA; 3 PRISE Center: Partnerships for Research in Implementation Science for Equity, University of California, San Francisco, CA USA; 4 Healthforce Center, University of California, San Francisco, CA USA; 5 Immigrant Health and Cancer Disparities Service, Department of Psychiatry and Behavioral Sciences, Hospital Medicine Service, Department of Medicine, Memorial Sloan Kettering Cancer Center, New York, NY USA; 6 Asian American Research Center on Health, San Francisco, CA USA; 7 Department of Medicine, University of California, Zuckerberg San Francisco General Hospital and Trauma Center, San Francisco, San Francisco, CA USA; 8 Department of Behavioral, Social, and Health Education Sciences, Rollins School of Public Health, Emory University, Atlanta, GA USA

**Keywords:** language concordance, provider non-English-language assessment, doctor-patient communication

## Abstract

**Background:**

Language concordance can increase access to care for patients with language barriers and improve patient health outcomes. However, systematically assessing and tracking physician non-English language skills remains uncommon in most health systems. This is a missed opportunity for health systems to maximize language-concordant care.

**Objective:**

To determine barriers and facilitators to participation in non-English language proficiency assessment among primary care physicians.

**Design:**

Qualitative, semi-structured interviews.

**Participants:**

Eleven fully and partially bilingual primary care physicians from a large academic health system with a language certification program (using a clinician oral proficiency interview).

**Approach:**

Interviews aimed to identify barriers and facilitators to participation in non-English language assessment. Two researchers independently and iteratively coded transcripts using a thematic analysis approach with constant comparison to identify themes.

**Key Results:**

Most participants were women (*N*= 9; 82%). Participants reported proficiency in Cantonese, Mandarin, Russian, and Spanish. All fully bilingual participants (*n*=5) had passed the language assessment; of the partially bilingual participants (*n*=6), four did not test, one passed with marginal proficiency, and one did not pass. Three themes emerged as barriers to assessment participation: (1) beliefs about the negative consequences (emotional and material) of not passing the test, (2) time constraints and competing demands, and (3) challenging test format and structure. Four themes emerged as facilitators to increase assessment adoption: (1) messaging consistent with professional ethos, (2) organizational culture that incentivizes certification, (3) personal empowerment about language proficiency, and (4) individuals championing certification.

**Conclusions:**

To increase language assessment participation and thus ensure quality language-concordant care, health systems must address the identified barriers physicians experience and leverage potential facilitators. Findings can inform health system interventions to standardize the requirements and process, increase transparency, provide resources for preparation and remediation, utilize messaging focused on patient care quality and safety, and incentivize participation.

**Supplementary Information:**

The online version contains supplementary material available at 10.1007/s11606-023-08354-6.

## INTRODUCTION

In the USA, almost twenty-six million individuals speak English less than very well (i.e., have limited English proficiency [LEP]).^1^ Individuals with LEP experience disparities in care, including decreased comprehension of medical diagnoses, poorer adherence to medication and lifestyle recommendations, increased medication complications, and decreased satisfaction with care compared to English-speaking individuals.^2–7^ Language concordant care, defined as when patients and their physicians communicate in the same language, can help bridge language barriers.^8–10^

Language-concordant care is associated with improved health outcomes for patients with LEP.^9,11–17^ A systematic review^9^ found that language-concordant primary care is associated with better patient access to and utilization of primary care, improved diabetes control and associated risk factors, and a higher likelihood that patients would receive and agree with diet and physical activity counseling compared to language discordant care. This review also found a positive impact on patient care experience and communication, with patients reporting higher satisfaction with care, better health education, enhanced privacy, enhanced communication, fewer lingering questions about care, and increased therapeutic alliance with physicians.

Yet physician use of semi-proficient non-English language skills can pose problems and risks to patient care. For example, care provided by partially fluent physicians, such as after completion of “medical Spanish” courses without subsequent assessment of their proficiency, can be as problematic as using ad hoc interpreters (i.e., family, friends, or untrained staff).^18,19^ Inadequate skills can lead to errors in communication and care, including more errors of omission and physician, rather than patient, centered communication.^7,19–21^

Thus, assessing physician non-English language proficiency and providing health system certification for such language use are crucial components to ensuring language access and providing high-quality care for patients with LEP. Despite this, physician non-English language skill use in clinical care is inconsistently assessed and tracked, representing a missed opportunity to maximize language-concordant care.^18^ The objective of this qualitative study was to understand clinician barriers to non-English language proficiency assessment and potential facilitators to increase clinician adoption of language certification.

## METHODS

### Recruitment Procedures

This qualitative study was part of the Language Access System Improvement (LASI) study, a previously described mixed-methods study in one health system.^22,23^ LASI evaluated the effects of simultaneously increasing access to professional interpreters and certifying bilingual clinicians’ language skills on communication and clinical outcomes in primary care.

As part of LASI, two researchers trained in qualitative methodologies (MW and SM) conducted semi-structured interviews with primary care clinicians in 2019. Participants were asked about their use of professional interpreters during clinical encounters and, if applicable, perceptions of the current language proficiency assessment. We invited potential participants via email, with the goal of interviewing approximately equal numbers of clinicians from the following groups: monolingual English, fully bilingual, and partially bilingual. We define fully bilingual as clinicians who reported their non-English language skills as “very good” or “excellent” and partially bilingual clinicians as those reporting “good” on the Interagency Language Reporting (ILR) scale.^24^ For the current study, we included only the interviews with fully and partially bilingual clinicians. Each interview lasted 30–45 min and was conducted in-person or via video conferencing according to the participant’s preference. Participants were provided with written information and then consented verbally prior to the interview. Once the two interviewers felt that no new concepts or topics were emerging, thematic saturation was reached, and no further interviews were conducted.^25^

### Non-English Language Proficiency Testing

The study health system uses the Clinician Cultural and Linguistic Assessment (CCLA) for non-English language proficiency assessment.^26,27^ To our knowledge, CCLA is the only validated clinician oral proficiency interview and has been disseminated in public, non-profit, and academic settings. It is designed to assess a clinician’s ability to communicate with patients with LEP in their preferred language. It is conducted via telephone and consists of both objective and subjective components. The objective assessment scores the clinician’s level of proficiency (grammatical, discourse, sociolinguistic, and strategic competence) and the subjective assessment includes scores on fluency, pronunciation, cultural proficiency, and customer service. Two qualified and trained raters score the examination from 1 to 100 with 80 as the threshold passing score.^27,28^

### Clinician Semi-structured Interview Guide

The semi-structured interview guide was created with input from LASI’s clinician, policy, and interpreter services stakeholders.^22^ If a participant reported full or partial proficiency in a non-English language, we asked for their general feelings about assessing clinicians’ non-English language proficiency, whether they had considered or participated in the assessment, prior experiences with testing, opinions on test characteristics and appropriateness with their patient population and for their clinical specialty, perceived barriers to testing, and facilitating factors that might increase testing adoption (see Appendix).

### Analysis

Audio recordings were transcribed verbatim following each interview. We used thematic analysis with constant comparison to analyze transcribed interviews.^29^ The research team (MEG, MW, SM, SP, JJ, LK) independently coded then jointly created an initial codebook of data-driven codes using three physician interviews chosen at random. Two researchers (MW and SP) then independently coded two additional transcripts to confirm the definitions and reliability of the codes. The research team met to reconcile codes and review additional emergent themes. Using the updated codebook, the two researchers independently re-coded all transcripts, using Dedoose (SocioCultural Research Consultants, Manhattan Beach, CA). We developed themes from grouped codes, further refining them through discussion and the use of constant comparison within and between codes to ensure themes accurately reflected the data.^29^

## RESULTS

### Physician Participants

Of the sixteen clinicians originally interviewed (15 physicians, 1 nurse practitioner) as part of the LASI study, eleven physicians reported full (*N*=5) or partial (*N*=6) proficiency in a non-English language and were included in this analysis. The majority were women (*N*= 9; 82%). Five participants reported proficiency in Mandarin, four in Spanish, and one each in Cantonese and Russian. All fully bilingual participants had taken and passed the proficiency test; of the partially bilingual participants, four did not test, one passed with marginal proficiency, and the last did not pass the proficiency test.

### Themes

Three themes emerged as barriers to proficiency testing, while four themes emerged as facilitators that could increase the adoption of testing.

#### Barriers to Proficiency Testing

##### Theme 1: Beliefs About the Negative Consequences (Emotional and Material) of Not Passing Test

Participants described concerns about not passing the test, such as potential feelings of loss or guilt about overusing their language skills. They additionally worried that not passing the test could threaten their personal and professional identity. Participants described uncertainty about what would happen if they did not pass the test and whether privileges would be revoked or whether remediation or retaking the test was possible.

Participants expressed fear of losing their privileges to communicate in a non-English language if they did not pass the proficiency test. This was regardless of whether participants were fully or partially bilingual and had taken the test or not. One participant noted, “I've been doing this all along. What a bummer it would be if that privilege got taken away from me. I definitely hesitated to do the training or to go through the certification.” (P1, bilingual, Spanish, passed). Another participant emphasized patient preference for language concordance and potential loss of patient-clinician rapport; “patients prefer that, even if I don't have perfect language skills...They feel more comfortable with that one-on-one interaction rather than having the third person...for most things, I'm fluent enough” (P5, partially bilingual, Mandarin, not tested).

Participants were also concerned that testing would reveal they were using a non-English language without adequate proficiency and potentially providing substandard quality care to patients. One participant noted, “I was a little bit nervous about [taking the test] because I was gonna feel guilty if it said I wasn’t [proficient], and then I had been using Spanish all these years” (P10, bilingual, Spanish, passed). This concern was expressed by both partially and fully bilingual participants.

Participants further described that failing the test would feel like judgement or criticism of their roles or capacity as physicians and that care would need to be taken to remind “you that failure is not actually the end or your patient care skills...I think it’s just some reassurance that this is not reflective of who you are as a human being.” (P3, partially bilingual, Russian, not tested). Among bicultural physicians, there was also the concern that failing the test would feel like a negative judgement of their cultural heritage. A self-identified Mexican-American participant noted “I took the language test as an intern, and I got a 79% which was a hit to my ego. I felt like a bad representative of my culture, especially with it being my first language.” (P11, partially bilingual, Spanish, tested marginal).

Participants expressed uncertainty about the possibility of remediation, whether there would be resources available to improve language skills, and the timeline to retake the test if desired. One participant stressed “I think if the medical center said, okay if you want to be proficient but you’re not quite proficient through our testing, let’s figure out alternative things, alternative ways. Either helping you, figuring out are there materials that can help you, helping you get to that proficiency.” (P5, partially bilingual, Mandarin, not tested).

##### Theme 2: Time Constraints and Competing Demands

Participants described prioritization of time and competing priorities as barriers to proficiency testing. Furthermore, the lack of a clear mandate to obtain certification led participants to prioritize other demands, such as clinical and administrative duties. As one participant described, “If it’s not really required in any way and there’s not really an incentive to do it, then why do it…[it] manages to slip to the bottom of the list every time if there’s not really a deadline or a payment or a requirement for it.” (P10, bilingual, Spanish, passed).

##### Theme 3: Challenging Test Format and Structure

Participants felt that the test was not representative of a patient-physician conversation, that the register and difficulty of the test were too high, and that the uniform content used in the testing was not applicable to their clinical practice. They further described a dislike of testing in general, which made language assessment more challenging.

Participants perceived that the test was not reflective of routine ambulatory clinical practice. They felt the test inhibited back-and-forth communication and the opportunity to clarify meaning and patient understanding. One participant described the one-way interaction via telephonic test as “speaking into the void.” Participants questioned whether the test was therefore truly the gold standard for proficiency, with one participant emphasizing, “It is awkward…It’s like a recording, you just speak into blank space. There are lots of nonverbal cues that you do when you are in an interaction with a patient that also matter, that help get your point across rather than just words.” (P5, partially bilingual, Mandarin, not tested).

Physicians additionally perceived the test to be hard, either from taking the test (even if they passed), starting the test (but not completing), or speaking to someone else about the test. Furthermore, participants expressed concern that the assessment was not reflective of the health literacy of patients they saw in clinical practice. One participant remembered taking the test and thinking, “Gosh, this is hard. And I don’t know if I’m going to pass, because I don’t use a lot of technical terms. A lot of my patients aren’t that educated. …We wanted sixth, seventh grade education level and bring it down to that level” (P6, bilingual, Cantonese, passed).

Participants, who were all general internists, additionally felt that uniform content that included specialized (such as describing HIV transmission in detail) or cross-specialty scenarios (with pediatric or gynecologic examples) was off-putting. One participant remembered, “I looked at some of the practice cases and they were so different from what we talk about in our visits…They might ask me about how to talk to a child or a pregnant woman…I’m like, ‘But that’s not what I’m going to talk about.’ Is it truly relevant to what I’m talking about every single day with patients?” (P3, partially bilingual, Russian, not tested).

Finally, participants expressed general reluctance to test, particularly in the context of feeling over-tested and the need to maintain regular health system and specialty training requirements. High proficiency speakers particularly felt that testing was unnecessary and onerous, with one participant explaining, “I was ambivalent because I hate taking tests and I didn’t want to do it. I know I have a high proficiency because I lived and studied in Asia.” (P2, bilingual, Mandarin, passed)

#### Facilitators to Increase Adoption of Assessment

##### Theme 1: Messaging Consistent with Professional Ethos

Physicians felt that appealing to professional identity or role, with an emphasis on providing patient-centered care, would be more effective in motivating physicians to test than punitive messaging or requirements. One physician emphasized “most providers care about their patients and they want to do right by the patient.” (P6, bilingual, Cantonese, passed). Participants preferred messaging that highlighted the importance of knowing physician language proficiency to ensure quality patient care.

##### Theme 2: Organizational Culture That Values and Incentivizes Certification

Participants expressed that an organizational culture that values and incentivizes certification, either with recognition, financial incentives, or support and resources to prepare for the test or remediation resources if they did not pass, as well as a clear mandate or policy for certification would promote increased participation in testing.

Participants believed that if the organization provided recognition that being certified matters, and that being bilingual is an asset, it would serve as an incentive to test. One partially bilingual physician noted, “if it was something that leadership and other people were putting out there, like this is a great thing that we should be doing, that would incentivize me, too.” (P11, partially bilingual, Spanish, tested marginal). Participants suggested that recognition could take the form of public acknowledgement of bilingual skills, through health system messaging about the importance and contribution of bilingual providers to patient care, or financial incentives to enable more physicians to prioritize the language proficiency test.

Suggestions for support also included clinical credit or blocking off time from clinical care to test, support to prepare for the test, and remediation resources if they did not pass. Partially bilingual participants, especially, expressed that opportunities for growth, via feedback on their proficiency after taking the test, or resources for improvement would increase testing. One participant explained it “would encourage me to take the test knowing that there’s something else on the other side where I might be able to get better and eventually pass as well.” (P4, partially bilingual, Mandarin, not tested).

Finally, physicians felt that the current policy for testing was unclear, with vague consequences for not testing. A partially bilingual physician said one potential motivator to testing “would be if there was a mandate that if you weren’t certified, that you had to use an interpreter. Which I think there is that expectation now, except I don’t think it’s followed, it’s not audited, and nobody really pays attention to that.” (P5, partially bilingual, Mandarin, not tested). Thus, a policy that was uniformly applied and understood throughout the organization would incentivize bilingual physicians to test their language proficiency.

##### Theme 3: Reassurance and Empowerment About Personal Language Proficiency

Participants described scenarios when passing or not passing the test could provide reassurance and empowerment about an individual’s language proficiency, including both not needing or needing to work with a professional interpreter.

Participants felt that passing the test provided personal reassurance that they could conduct a clinical visit with a patient with LEP without the assistance of an interpreter. One participant said, “I do want a reassurance that I’m able to provide the proficient amount of Mandarin. So I’m not putting my patient at a disadvantage by just getting by.” (P8, bilingual, Mandarin, passed). Similarly, for physicians who did not speak a trainee’s non-English language, knowledge that a trainee was certified served as reassurance that trainees could conduct clinical visits with patients in that language without the use of an interpreter, “I like it because I can trust when a resident is certified, I can trust the information that they’ve received from the patient as much as somebody with English-English concordance.” (P7, partially bilingual, Spanish, not tested).

Conversely, physicians reported that not passing the test could provide a sense of relief in certain circumstances where they doubted the extent of their language proficiency. This was particularly true when there was patient preference or pressure for the provider to “get by” with existing language skills. As one participant noted, “I think I was actually relieved that I failed [the test] so then I could feel more like, yeah, I really do need an interpreter with me all the time. It empowered me to still use one.” (P9, partially bilingual, Mandarin, did not pass).

Participants described that reassurance that they could still use an interpreter, even if they were certified in a specific language, could serve as an incentive to test. Bilingual physicians described that while they feel confident in their language skills in most circumstances, they understand that language proficiency is a spectrum and often situational. Therefore, in certain circumstances, such as in complex discussions or goals of care conversations, some still preferred to use an interpreter or have one available for back-up, since, as a participant explained, “I would have an interpreter present because I think the threshold to pass the exam is not necessarily the threshold to have a conversation of that depth [with] a lot of patients.” (P11, partially bilingual, Spanish, tested marginal).

##### Theme 4: Individuals Championing Certification

Participants suggested that messaging from trusted individuals could also be leveraged to increase adoption in the future, “I think a testimonial or a video from somebody who they might recognize to say why it’s important, what that does for patient care…I think the way it was messaged…it almost seemed like it could be punitive to the providers. And so, maybe different messaging. Hearing from a provider, this is why it’s important for patient care, might get more buy-in.” (P1, bilingual, Spanish, passed). Participants stressed that champions or role models could encourage proficiency testing; participants could further query these individuals regarding test format, structure, and difficulty level.

## DISCUSSION

Our study identified salient barriers and facilitators to language proficiency assessment in one academic health system. While some of the barriers were specific to the CCLA test format and structure, others related to emotionally charged concerns about the consequences of failing the test and how to balance testing with competing demands and time constraints. Importantly, participants valued certification and the reassurance it provided about using (or not using) their non-English language proficiency skills or trusting trainees’ bilingual language skills.

To ensure quality language-concordant care for patients with LEP, we must increase non-English language assessment and certification of clinicians using validated tools. The complexity and nuance of the decision to undergo a non-English language proficiency assessment were reflected in the identified themes. Health systems can use these findings to develop targeted interventions to start or improve a language assessment and certification process in their unique settings, prioritizing the most salient barriers and enablers in their contexts and implementing intervention strategies and policies that address identified barriers/enablers a priori (see Table 1).

**Table 1:** Themes, Descriptions, and Potential Health System Intervention Components

Theme	Description	Potential intervention components
Belief about the negative consequences (emotional and material) of not passing test	Concerns about not passing the test, such as feeling guilty about overusing language skills; worry that not passing would threaten personal or professional identity; uncertainty about consequences of not passing the exam, such as loss of privileges or opportunity to retake the test.	• Messaging incorporating champions and addressing the emotional barriers to assessment and the potential perceived threats to personal or professional identity • Transparency about assessment format, structure, and scoring, as well as opportunities for retaking the assessment • Resources to support improvement to reach passing threshold
Time constraints and competing demands	Prioritization of time and competing priorities as barriers to proficiency testing.	• Schedule time for new hires and existing employees to take assessment • Incorporate assessment into clinical schedule • Create and communicate clear policy for certification
Challenging test format and structure	Test not representative of a patient-clinician conversation; test register and difficulty too high; uniform content not applicable; dislike of testing in general.	• Informational materials about assessment format, structure, and scoring • Consider alternative assessment options such as validated approaches to direct observation • Recruit champions and opinion leaders to reinforce importance of assessment
Messaging consistent with professional ethos	Recommendations to appeal to clinicians’ professional identity or role, emphasizing patient-centered care, rather than punitive messaging.	• Messaging campaigns to encourage certification, emphasizing quality patient care
Organizational culture that values and incentivizes certification	Need for recognition, financial incentives, or support and resources; a clear mandate or policy for certification.	• Financial incentives and rewards to get certified and to see patients in non-English language once certified • Assessment guidelines for new bilingual hires and existing employees, with standardized procedures • Dashboard of clinicians’ non-English skills and certification which is made available to patients and employees • Visible recognition for certified bilingual clinicians, for example, an ID sticker, listing on a website
Reassurance and empowerment about personal language proficiency	Passing the test could serve as reassurance not to use an interpreter in clinical encounters or for a clinical supervisor to trust a trainee’s language skills; not passing the test could serve as reassurance to use an interpreter; reassurance that it is ok to use an interpreter when clinician feels one is needed, even after passing the test.	• Training sessions and guidance on appropriate use of interpreters • Messaging campaigns to encourage certification, emphasizing quality patient care • Guidance on accessing professional interpreters, regardless of certification status • Educational sessions about certification and ensuring language access
Individuals championing certification	Messaging from trusted individuals.	• Recruit champions and opinion leaders to reinforce importance of assessment • Messaging campaigns to encourage certification, emphasizing quality patient care

Certification may be most useful in helping partially bilingual providers make the decision to use or not use an interpreter.^26,31,32^ Participants in our study described that the test served as reassurance regarding their language skills, whether or not they passed. While imperfect, testing takes some of the guesswork out of whether partially bilingual physicians should be using their skills in clinical care. Partially bilingual individuals may inappropriately use their language skills when they miscalculate the complexity or risk for miscommunication, overestimate the patient’s English proficiency, or miscalculate their own non-English proficiency.^33^ This may be particularly true after short medical non-English language courses which may give clinicians a false sense of fluency.^19,32,34,35^ Formal assessment may help clinicians take a realistic view of their own language proficiency skills.^19,32^

Health systems can take some immediate steps to increase non-English language proficiency testing [Table 1]. They can clarify requirements and mandates for assessment and standardize the process by encouraging assessment when people are hired in the organization or with regular privilege renewal processes. This would automate assessment and further demonstrate to patients and clinicians that health systems value certification. Health systems can provide additional information about the format, structure, and scoring and clearly define the next steps if clinicians do not pass the assessment. Health systems can share resources to help clinicians with marginal language proficiency improve their skills. They can also provide guidance as to how the assessment results should be interpreted, and that passing the assessment does not preclude using an interpreter. Increasing language access for patients with LEP is an unfunded mandate, yet health systems must balance the cost of interpreters and certification programs with the economic consequences of miscommunication, such as through malpractice lawsuits or other legal action*.*^30^

Messaging regarding testing should focus on providing quality care for patients, consistent with a clinician’s professional ethos. Campaigns should utilize positive messaging and incorporate champions and clinical leaders, rather than messaging focused on the punitive aspects of assessment (i.e., loss of privileges). Results indicate a need for sensitivity to the emotional components associated with assessment, as identified by bilingual and bicultural clinicians in this study. Health system messaging should focus on the situational and contextual nature of proficiency and clarify that even certified clinicians may need to use interpreters in certain circumstances. Further work can explore whether a minimal set of barriers needs to be addressed to improve physician certification or a more holistic approach is needed (e.g., addressing the “emotionally charged” issues as well).

As there are various language proficiency tests available, health systems should consider whether a given test has been validated and serves their needs; and then report proficiency in a standardized way to ensure consistency and provide guidance to physicians and patients. While targeted interventions may increase assessment participation and overcome some barriers, health systems may also need new forms of assessment. One promising alternative is to use direct observation of language skills during a clinical visit to certify bilingual physicians. Direct observation could potentially overcome some of the inherent difficulties of current testing identified by our participants (i.e., challenging test format and structure, time constraints, and competing demands). However, it will be important to use a validated tool for direct observation to ensure its use in clinical settings is accurately assessing language proficiency; such a tool has been developed and tested for reliability by members of our team and is currently being validated.^36^

Our study has limitations. It was conducted in one academic, outpatient clinical setting with good access to interpreter services, which may have affected the physicians’ calculus to test versus use professional interpreters. Furthermore, while our site used the CCLA, other tests are available, and no gold standard exists. Therefore, the perceptions of test characteristics in our sample may not be generalizable to other assessments. However, the CCLA is widely used, and participant experiences described in this study may further inform a health systems’ choice of test, decision to adopt the CCLA, or to incorporate alternative forms of assessment such as direct observation of clinical language proficiency when an assessment tool is validated and available. Finally, our study only included general internists; other barriers may exist for physicians in other specialties and settings or for other health professionals.

Barriers and facilitators to testing non-English-language proficiency are complex. Some are procedural, yet many more relate to individual motivation and social and environmental context. These will require targeted multi-component interventions or changes to current assessment practices. To ensure adequate language access for patients with LEP, in addition to making professional interpreters more widely available and encouraging their use, health systems will need to ensure quality language concordant care.

### Supplementary Information

ESM 1 (DOCX 24.8 kb)

## Data Availability

The datasets used and/or analyzed during the current study are available from the last author on reasonable request.

## Abbreviations

CCLA: Clinician Cultural and Linguistic Assessment
ILR: Interagency Language Reporting Scale
LEP: Limited English Proficiency
LASI: Language Access System Improvement Study
