# Supplementary material for: Language-Concordant Care: a Qualitative Study Examining Implementation of Physician Non-English Language Proficiency Assessment
Source: J Gen Intern Med. 2023 Aug 24;38(14):3099–106. doi: 10.1007/s11606-023-08354-6 (PMC10651569; doi:10.1007/s11606-023-08354-6)
Supplement: Supplementary file 1 — (DOCX 24.8 kb) [file 11606_2023_8354_MOESM1_ESM.docx]

**Research question**

1. How can we improve UCSF language access systems through bilingual clinician certification program

**Interviewees to target – 5+ from each group**

**Group 1**: Bilingual PCPs: Ask about testing experiences

**Group 2**: Partially bilingual PCPs: Ask about testing experience

**FULLY BILINGUAL PCP [Visit conducted in a non-English language that the doctor and patient both speak fluently] and PARTIALLY BILINGUAL PCP [Visit where doctor and patient are language DISCORDANT]**

[Relevant excerpt from interview guide]

1. Tell me what you know about testing clinicians for their proficiency in another language.
   1. How do you feel about it?
   2. Would you consider being tested in your language proficiency in a language other than English? Why would you or would you not?
   3. Have you been tested? Tell me about your experience with that.
   4. In your opinion, does the test use the kind of language you use with patients? Was it more formal? Less formal?
   5. What do you think would encourage more clinicians to be tested?
   6. What do you think might dissuade clinicians from getting testing?
   7. What do you think about the possibility of having your language skills observed and assessed during a clinical visit instead of taking a separate test? Would you participate in this type of observation? What would encourage you to participate? What would dissuade you from participating? (Probes: how many observations? Why? What range of skills or contexts to be observed?)
